# The CBP60b-14-3–3 functional module regulates immunity in arabidopsis

**DOI:** 10.3389/fpls.2026.1916745

**Published:** 2026-08-05

**Authors:** Lu-Shen Li, Lin Ma, Yun-Xia Chen, Sha Li

**Affiliations:** 1 College of Agriculture and Biology, Liaocheng University, Liaocheng, China; 2 State Key Laboratory of Wheat Improvement, College of Life Sciences, Shandong Agricultural University, Tai’an, China; 3 Frontiers Science Center for Cell Responses, College of Life Sciences, Nankai University, Tian’jin, China

**Keywords:** 14-3-3, calmodulin-binding protein 60b (CBP60b), plant immunity, protein interaction, transcription

## Abstract

Transcriptional reprogramming is a critical process in plant immune responses, in which Calmodulin-Binding Protein 60b (CBP60b), a plant-specific atypical transcription factor family, plays an essential role. However, how its transcriptional activity is regulated during immunity remains unclear. Here, we report that 14-3–3 proteins, conserved scaffolding proteins in eukaryotes, interact with CBP60b in a calcium-dependent manner and enhance its transcriptional activity on downstream genes. The 14-3–3 variant deficient in calcium binding, 14-3-3λ^G216A^, disrupted the interaction with CBP60b and failed to fully complement the pathogen-sensitive phenotype of the *14-3-3λ;κ* mutant. Furthermore, we show that the pathogen-induced transcriptional upregulation of *14-3-3λ* and *14-3-3κ* is dependent on CBP60b. Finally, genetic evidence demonstrates that 14-3-3s are required for CBP60b function. Collectively, our findings reveal that CBP60b and 14-3–3 proteins form an interactome-mediated positive feedback loop to orchestrate immune responses in a calcium-dependent manner.

## Introduction

As sessile organisms, plants constantly face the challenge of recognizing pathogens and activating immune responses in a timely and appropriately manner. The plant innate immune system is initiated by plasma membrane-localized pattern recognition receptors (PRRs) and intracellular nucleotide-binding domain and leucine-rich repeat-containing receptors (NLRs) (Weralupitiya et al., 2024), which activate PTI and ETI signaling cascades, respectively, leading to immune activation and defense against pathogen invasion (Zhou and Zhang, 2020; Ngou et al., 2022; Zhang et al., 2023; Weralupitiya et al., 2024). Although PRRs and NLRs possess distinct biochemical activities and are activated through relatively independent mechanisms (Weralupitiya et al., 2024), their downstream immune outputs are strikingly similar, including mitogen-activated protein kinase (MAPK) cascades, reactive oxygen species (ROS) bursts, defense hormone production, Ca²^+^ influx, and transcriptional reprogramming (Zhang et al., 2023; Weralupitiya et al., 2024). However, ETI typically triggers stronger immune responses and often induces hypersensitive response (HR), a form of rapid localized cell death (Zhou and Zhang, 2020; Ngou et al., 2022). Recent studies have revealed that the previously considered largely independent and parallel PTI and ETI pathways mutually enhance each other in a synergistic manner, enabling plants to mount sustained and robust immune outputs (Lozano-Durán et al., 2023).

Ca²^+^ influx upon pathogen infection has long been considered a critical step in initiating plant immune responses (Jiang and Ding, 2023; Wang and Luan, 2024). Ca²^+^ channels in PTI are typical ion channels that contain multiple transmembrane domains, including cyclic nucleotide-gated channels (CNGCs) (Tian et al., 2019), hyperosmolarity-induced Ca²^+^ channels (OSCAs) (Thor et al., 2020), and glutamate receptor-like channels (GLRs) (Bjornson et al., 2021). In ETI, two classes of NLRs—CNLs and RNLs—are activated by pathogens and assemble into multimeric resistosomes that mediate Ca²^+^ influx (Huang et al., 2023). Thus, Ca²^+^ influx during immune responses represents a convergent output of immune initiation mediated by both cell surface and intracellular immune receptors (Wang and Luan, 2024). However, distinct Ca²^+^ signals trigger divergent downstream signaling pathways with varying strengths. Therefore, decoding the spatiotemporally dynamic Ca²^+^ signals in cellular immune responses and elucidating the underlying molecular mechanisms are of great significance (Jiang and Ding, 2023).

14-3–3 proteins are highly conserved eukaryotic regulators that play critical roles in cellular signal transduction (Denison et al., 2011; Lozano-Durán and Robatzek, 2015; Camoni et al., 2018). They typically form homo- or heterodimers via their N-terminal domains, with each monomer capable of binding a target protein (Sheikh et al., 2024). This allows 14-3–3 proteins to function as molecular scaffolds that mediate protein–protein interactions, thereby modulating the activity, stability, subcellular localization, and protein complex status of their clients (Sheikh et al., 2024). Most known target proteins require phosphorylation to associate with 14-3–3 proteins and, with some exceptions, contain conserved recognition motifs (Lozano-Durán and Robatzek, 2015; Sheikh et al., 2024).

In Arabidopsis, 14-3-3λ/GRF6 and 14-3-3κ/GRF8 have been reported to participate in immune regulation (Lozano-Durán and Robatzek, 2015; Sheikh et al., 2024). They interact with multiple RLCKs and MAPKKKs. MAPKKK5 undergoes intramolecular interaction between its N- and C-terminal regions, which restricts access of RLCKs to the C terminus. 14-3–3 proteins bind the C terminus of MAPKKK5 and facilitate the association of RLCKs with this region, leading to MAPKKK5 phosphorylation and subsequent MAP kinase cascade activation (Dong et al., 2023). The *Pseudomonas syringae* effector HopM1 targets 14-3-3κ and promotes its degradation, thereby suppressing PTI signaling (Nomura et al., 2006). Additionally, 14-3-3λ has been shown to interact with the NLR protein RPW8 and contribute to pathogen resistance (Yang et al., 2009). Collectively, these findings demonstrate that 14-3-3λ and 14-3-3κ are involved in PTI, ETS, and ETI, and serve as critical regulators of plant immune responses. Beyond Arabidopsis, studies in tomato, rice, pepper, and tobacco have also highlighted 14-3–3 proteins as a major hub for plant immunity (Oh et al., 2010; Oh and Martin, 2011; He et al., 2012; Teper et al., 2013; Deb et al., 2019; Sheikh et al., 2023). Furthermore, 14-3-3λ and 14-3-3κ contain EF-hand motifs and have been shown to bind Ca²^+^ in response to salt stress, thereby modulating the kinase activities of SOS2 and PKS5 (Yang et al., 2019). However, whether their immune-related functions are regulated by Ca²^+^, and how their expression is controlled at the transcriptional level, remain to be explored.

Transcriptional reprogramming is central to plant immune responses (Huang et al., 2023). Accumulating evidence demonstrates the crucial role of CBP60 in immune transcriptional reprogramming (Qin et al., 2018; Huang et al., 2020; Kim et al., 2022; Li et al., 2024). As plant-specific atypical transcription factors, the CBP60 family in Arabidopsis consists of eight members, which can be phylogenetically classified into four clades: CBP60a, CBP60b–f, CBP60g, and SARD1 (Zheng et al., 2021; Li et al., 2024). CBP60g and SARD1 function redundantly in regulating the expression of immune-related genes involved in PTI, ETI, SA, and SAR signal pathway (Wang et al., 2011; Sun et al., 2015), and also play critical roles in positive feedback loops regulating SA and NHP biosynthesis (Huang et al., 2020). CBP60b, a key transcription factor in immunity (Huang et al., 2021; Li et al., 2021, Li et al., 2024), not only regulates a set of immune genes overlapping with those controlled by CBP60g and SARD1, but also directly binds to the promoters of *CBP60g* and *SARD1* to modulate their expression. CBP60b may serve as a target of pathogen effectors and is subject to surveillance by NLR proteins; its loss leads to EDS1-PAD4– and NDR1-dependent autoimmunity (Huang et al., 2021; Li et al., 2021). Recent studies suggest that the CBP60b–f clade represents the ancestral form of CBP60s during land plant lineage evolution (Zheng et al., 2021; Li et al., 2024), and that the CBP60b-centered immune regulatory mechanism is conserved in higher plant lineage (Li et al., 2024). Moreover, calmodulin (CaM) binds to CBP60b–f and CBP60g in a Ca²^+^-dependent manner to modulate their function during immune responses (Reddy et al., 2002; Wang et al., 2009; Li et al., 2024), indicating that their immune activities are regulated by Ca²^+^ signaling.

Although CBP60b is known to regulate a set of immune-related genes by binding conserved promoter motifs, how its transcriptional activity is modulated during immune responses remains unclear. Here, we demonstrate that 14-3–3 proteins interact with CBP60b and enhance its transcriptional activity. A calcium-binding–deficient variant, 14-3-3λ^G216A^, disrupted the interaction with CBP60b and failed to fully complement the immune defects of the *14-3-3λ;κ* mutant, indicating that the immune function of 14-3-3s are calcium-dependent. Furthermore, we show that CBP60b directly regulates the expression of *14-3-3λ* and *14-3-3κ* during immunity, and genetic evidence demonstrates that 14-3-3s are required for CBP60b function.

## Materials and methods

### Plant materials and growth conditions

*Arabidopsis thaliana* Columbia-0 was used as the wild type. Plant materials including *cbp60b-1*, *eds1;pad4*, *cbp60b;eds1;pad4*, *14-3-3λ;κ*, *CBP60b-GFP COM* line, and *CBP60b OE-1/-2* lines were described previously (Zhou et al., 2014; Li et al., 2021). Plants were grown in a growth chamber at 22 °C under LD condition (16h light/8h dark) or SD condition (12h light/12h dark). Arabidopsis plants were transformed and selected as described (Li et al., 2021).

### Plasmid construction

Gateway™ technology (Invitrogen) was used for vector construction unless specified. Entry vectors were generated in pENTR/D/TOPO (Invitrogen). Previously reported vectors included *CBP60b* entry and *35S:U1-70K-mRFP* (Li et al., 2021). Entry clones for *14-3-3λ* (primers LW56/LW57) and *14-3-3κ* (P1216/P1217) were recombined via LR reactions into destination vectors to generate *35S:nEYFP-CBP60b*, *35S:14-3-3λ-cEYFP*, *35S:14-3-3κ-cEYFP*, *UBQ10:14-3-3λ-GFP*, and *UBQ10:14-3-3κ-GFP*. For pull-down assays, coding sequences of *14-3-3λ* (P2577/P2578), *14-3-3κ* (P2529/P2580), *MBP-CBP60b* (W1822/W1823), and *Flag-CBP60b* (LW9/LW10) were amplified and assembled using the pEASY-Uni Seamless Cloning Kit. The fragments of 14-3-3λ and 14-3-3κ were recombined into *Bam*HI/*Xho*I digested *pET-32a* to generate *His-14-3-3λ/κ*; *MBP-CBP60b* and *Flag-CBP60b* fragments were recombined into *Bam*HI/*Sal*I digested *pMAL-C2X* and *Bam*HI/*Stu*I digested *pHBT-Flag*, respectively. Mutagenesis primers: LW52/LW53 for *14-3-3λ^G216A^*, LW11/LW12 for *CBP60b^S568A^*, LW24/LW25 for *CBP60b^S455A^*, LW32/LW33 for *CBP60b^S60A^*, LW20/LW21 for *CBP60b^S103A^*, and LW22/LW23 for *CBP60b^S220A^*.

For dual luciferase reporter assays in Arabidopsis protoplasts, the *35S:CBP60b-GFP* vector was described previously (Li et al., 2021). Promoter fragments of *14-3-3λ* (LW45/LW46) and *14-3-3κ* (LW51/LW44) were inserted into *Kpn*I/*Sal*I digested pGreen II-0800 (Li et al., 2021) using the pEASY-Uni Seamless Cloning Kit to generate LUC reporter constructs.

### BiFC assays

The vectors *35S:14-3-3λ-cEYFP*, *35S:14-3-3λ^G216A^-cEYFP*, 3*5S:14-3-3κ-cEYFP*, *35S:nEYFP-CBP60b*, and *35S:U1-70K-mRFP* were transformed into *A. tumefaciens* GV3101. Bacteria were harvested, resuspended, and co-infiltrated with P19 into *N. benthamiana* leaves. After 48 h, eYFP signals were examined in U1-70K-mRFP-positive epidermal cells by confocal microscopy. Three biological replicates were performed per BiFC combination.

### Pull-down assays

The *His-14-3-3λ/κ* and *MBP-CBP60b* were expressed in BL21 (DE3) strain and purified using Ni-NTA agarose (Molecular Cloning Laboratories) or amylose resin (New England Biolabs), respectively. Purified proteins were incubated with amylose magnetic beads (PuriMag Pro) in binding buffer (20 mM Tris-HCl, 0.2 M NaCl, 1mM EDTA, 1mM DTT, 1mM CaCl_2_, pH = 7.4) at 4 °C for 2 h. After five washes with binding buffer, the proteins were analyzed by using immunoblotting with anti-MBP (1:5, 000) and anti-His (1:5, 000) antibodies (Transgen Biotech).

### Co-immunoprecipitation assays

Protoplasts were isolated from 4-week-old Arabidopsis plants. *pHBT-Flag-CBP60b* was co-transfected with *UBQ10:GFP* or *UBQ10:14-3-3λ/κ-GFP* into protoplasts. Total protein was extracted in buffer (50 mM Tris-HCl, pH=7.5, 150 mM NaCl, 1 mM EDTA, 1% Triton X-100, protease inhibitors). For anti-Flag immunoprecipitation, extracts were incubated with Anti-Flag Nanobody Magarose Beads (AlpalifeBio) at 4 °C for 2 h. Beads were washed five times with wash buffer (50 mM Tris-HCl, pH=7.5, 150 mM NaCl, 1 mM EDTA), boiled in loading buffer, and resolved by 7.5% SDS-PAGE. Flag-CBP60b and GFP-tagged proteins were detected using anti-Flag and anti-GFP antibodies (Transgen Biotech, 1:5, 000 each).

### Dual luciferase reporter assays in Arabidopsis protoplasts

Dual luciferase reporter assays were performed as described (Li et al., 2021). Briefly, Arabidopsis protoplasts were co-transformed with effector and reporter constructs via the PEG method (Yoo et al., 2007). Luciferase activity was measured using a Double-Luciferase Reporter Assay Kit (TransGen Biotech), and the ratio of promoter-driven LUC to 35S-driven Renilla LUC was calculated to determine transcriptional activity (Li et al., 2021).

### Pathogen inoculation and bacterial growth assays

Pathogen growth and inoculation were performed as described (Li et al., 2021). In brief, *Pto* DC3000 was cultured in King’s B medium (protease peptone, 10 mg/ml; glycerol, 15 mg/ml; K_2_HPO_4_, 1.5 mg/ml; MgSO_4_, 5 mM, pH 7.0) containing 12.5 mg/ml rifampicin. Five-week-old plants (SD conditions) were inoculated by infiltrating a bacterial suspension (OD600 = 0.0001 in 10 mM MgCl_2_) into mature leaves using a needleless syringe.

### RT-PCRs and RT-qPCRs

Total RNA was extracted from the 5^th^-6^th^ true leaves of 5-week-old plants (SD condition) using an Ultrapure RNA Kit (Cwbiotech). Reverse transcription and qPCR were performed with ReverTra Ace qPCR RT Master Mix (Toyobo) and SYBR Green master mix on an ABI QuantStudio™ 6 Flex. Each experiment included three to four biological replicates. *GAPDH* served as an internal control. Primers are listed in **Supplementary Table 1**.

### ChIP

ChIP assays were performed with the EpiQuik Plant ChIP kit (Epigentek) using 1.0 g of leaf tissue from WT and CBP60b-GFP plants *Pto* DC3000 inoculated for 24 h (Li et al., 2021). Chromatin was immunoprecipitated with anti-GFP antibody. Primers for qPCR are listed in **Supplementary Table 1**. Three biological replicates were performed.

### Fluorescence imaging

CBP60b-GFP fluorescence was imaged using a Zeiss LSM880 confocal microscope (excitation/emission: 488/513 nm).

### Accession numbers

TAIR accession numbers for the Arabidopsis genes used are provided below: AT5G10450 for *14-3-3λ*, AT5G65430 for *14-3-3κ*, AT5G57580 for *CBP60b*, AT5G26920 for *CBP60g*, AT1G73805 for *SARD1*, AT3G48090 for *EDS1*, AT3G52430 for *PAD4*, AT1G74710 for *SID2*, AT2G14610 for *PR1*, AT3G57260 for *PR2*, and AT3G04120 for *GAPDH*.

## Result

### 14-3-3λ and 14-3-3κ interact with CBP60b

**Figure 1:**
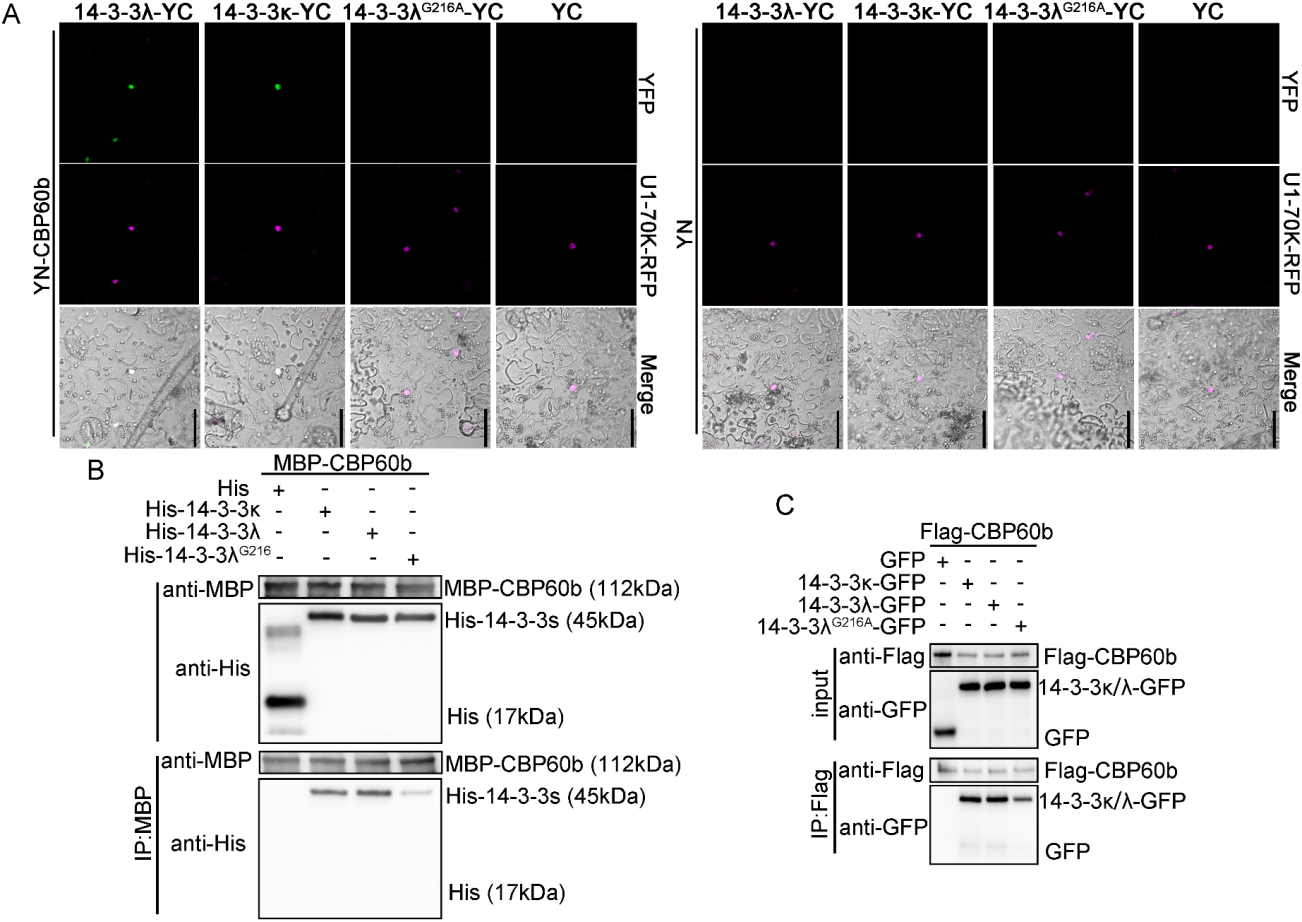
14-3-3λ and 14-3-3κ physically interact with CBP60b. **(A)** BiFC assays showing the interaction between 14-3-3s-cEYFP (14-3-3s-YC) and nEYFP-CBP60b (YN-CBP60b). U1-70K-mRFP was used to label the nucleus and to mark transformed cells. Bars = 50 μm. **(B)** Pull-down showing that 14-3-3s interact with CBP60b. Input and output were analyzed with anti-His and anti-MBP antibodies. **(C)** Co-immunoprecipitation of CPK4/5/6/11 with CBP60b in planta. Flag-CBP60b fusion protein was co-expressed with GFP or 14-3-3s-GFP in Arabidopsis protoplasts. Protein extracts were incubated with anti-FLAG magnetic agarose beads. Input and immunoprecipitates were analyzed by immunoblotting using anti-FLAG and anti-GFP antibodies.

14-3-3λ and 14-3-3κ, as members of the well-studied 14-3–3 scaffold protein family, play important roles in immunity (Sheikh et al., 2024). To investigate whether 14-3-3s regulate CBP60b-mediated immune responses, we first verified whether they directly interact. Bimolecular fluorescence complementation (BiFC) assays revealed clear interaction signals between CBP60b and 14-3-3λ and 14-3-3κ in the nucleus (**Figure 1A**). Subsequently, an *in vitro* pull-down assay demonstrated that MBP-CBP60b pulled down His-14-3-3λ/κ but not the His control (**Figure 1B**). Finally, co-immunoprecipitation (Co-IP) assays in leaf protoplasts further confirmed these findings (**Figure 1C**).

14-3-3λ Gly216 residue is a critical site for Ca²^+^ binding (Yang et al., 2019). To determine whether the interaction between 14-3-3s and CBP60b is Ca²^+^-dependent, we performed interaction assays. In BiFC assays, almost no fluorescence signal was detected in the nucleus for the 14-3-3λ^G216A^ in combination with CBP60b (**Figure 1A**), and both pull-down and Co-IP assays revealed reduced, but not abolished, binding of 14-3-3λ^G216A^ to CBP60b (**Figures 1B, C**). Together, these results indicate that Ca²^+^ mediate the interaction between 14-3-3s and CBP60b.

14-3-3-interacting proteins often require prior phosphorylation. To identify potential 14-3–3 binding sites on CBP60b, we analyzed the CBP60b protein sequence using 14-3-3-Pred (https://www.compbio.dundee.ac.uk/1433pred) (Madeira et al., 2015). Serine residues with the highest prediction scores (Consensus cut-off > 0.5) were mutated to alanine to assess their effects on the interaction (**Supplementary Figure 1A**). The results showed that these serine mutations still exhibited clear fluorescence signals with 14-3-3λ in the nucleus, indistinguishable from wild-type CBP60b (**Supplementary Figure 1B**), indicating that these predicted sites do not affect the interaction between CBP60b and 14-3-3λ.

### 14-3–3 enhances the transcriptional activity of CBP60b

**Figure 2:**
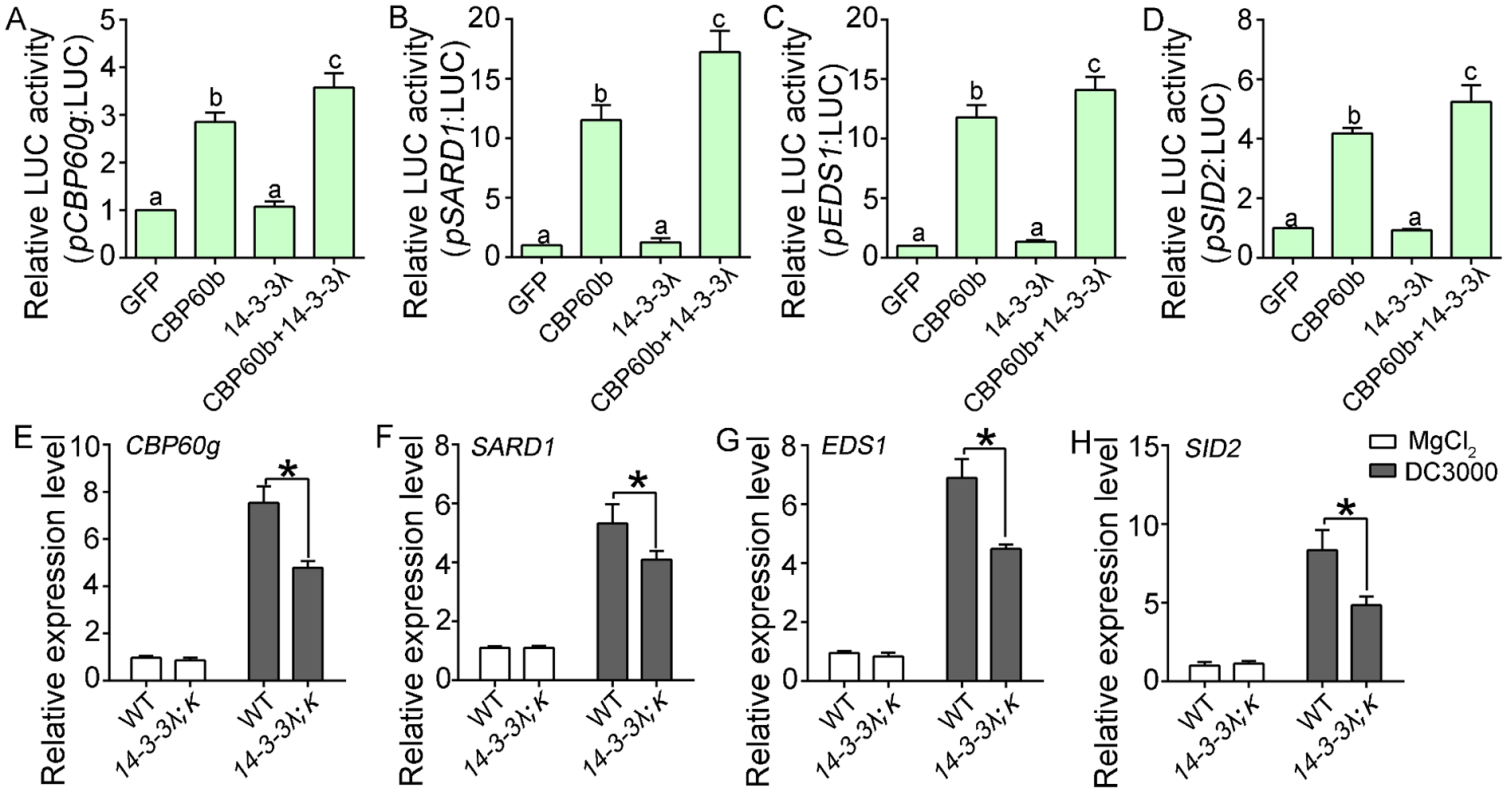
14-3-3s are essential for the transcriptional activity of CBP60b. **(A–D)** LUC activity of *proCBP60g:LUC* **(A)**, *proSARD1:LUC* **(B)**, *proEDS1:LUC* **(C)**, and *proSID2:LUC* **(D)** co-transfected with *35S:GFP* (GFP), *35S:CBP60b-GFP* (CBP60b), *35S:14-3-3λ-GFP* (14-3-3λ), or both *35S:CBP60b-GFP* and *35S:14-3-3λ-GFP* (CBP60b+14-3-3λ). Values are means ± SE (n = 3). Different letters indicate significantly different groups (1-way ANOVA and Tukey’s multiple comparison test; P < 0.05). **(E–H)** Relative transcript levels of *CBP60g* **(E)**, *SARD1* **(F)**, *EDS1* **(G)**, and *SID2* **(H)** in wild type (WT) and *14-3-3λ;κ* at 24 h after MgCl_2_ or *Pto* DC3000 treatment. Data are means ± SE (n = 3). Asterisks indicate significant difference (*t* test; P < 0.05).

14-3–3 proteins function as molecular scaffolds that modulate the activity, turnover, subcellular localization, and protein complex status of their target proteins (Sheikh et al., 2024). To investigate the role of 14-3-3s in regulating CBP60b function, we performed dual luciferase (LUC) reporter assays. It has been previously reported that CBP60b activates reporter activity driven by promoters of *CBP60g*, *SARD1*, *EDS1*, and *SID2* (Li et al., 2021). We found that co-expression of 14-3-3λ with the *promoter:LUC* reporter and CBP60b constructs enhanced CBP60b-induced LUC activity driven by *CBP60g*, *SARD1*, *EDS1* or *SARD1* promoters (**Figures 2A–D**), indicating that 14-3-3λ potentiates the transcriptional activity of CBP60b. To test whether this process is Ca^2+^-dependent, we used the calcium channel inhibitor LaCl_3_. The results showed that LaCl_3_ treatment significantly reduced CBP60b-induced LUC activity driven by the *SARD1* and *EDS1* promoters (**Supplementary Figure 2**), likely due to impaired binding between CBP60b and CaM (Li et al., 2024) upon LaCl_3_ treatment. Concurrently, we found that 14-3-3λ failed to enhance CBP60b transcriptional activity, suggesting that the 14-3-3λ mediated promotion of CBP60b activity is Ca^2+^-dependent. To further corroborate this finding, we examined the expression of CBP60b downstream genes in wild-type and *14-3-3λ;κ* mutant following *Pseudomonas syringae pv. tomato* DC3000 (*Pto* DC3000) inoculation. The transcript levels of *CBP60g*, *SARD1*, *EDS1*, and *SID2* were significantly reduced in the *14-3-3λ;κ* mutant upon pathogen challenge (**Figures 2E–H**), consistent with the above results.

Furthermore, we introduced CBP60b-GFP from the complementation lines (Li et al., 2021) into the *14-3-3λ;κ* mutant through genetic crossing. CBP60b is localized in the nucleus in the *14-3-3λ;κ* mutant, similar to its localization in wild-type background (**Supplementary Figures 3A, B**). Moreover, the protein accumulation of CBP60b-GFP in the *14-3-3λ;κ* mutant following pathogen inoculation was comparable to that in the wild-type background (**Supplementary Figure 3C**). These results indicate that 14-3–3 proteins do not affect the localization or protein accumulation of CBP60b.

### Gly216 is essential for 14-3-3λ to respond to pathogens.

**Figure 3:**
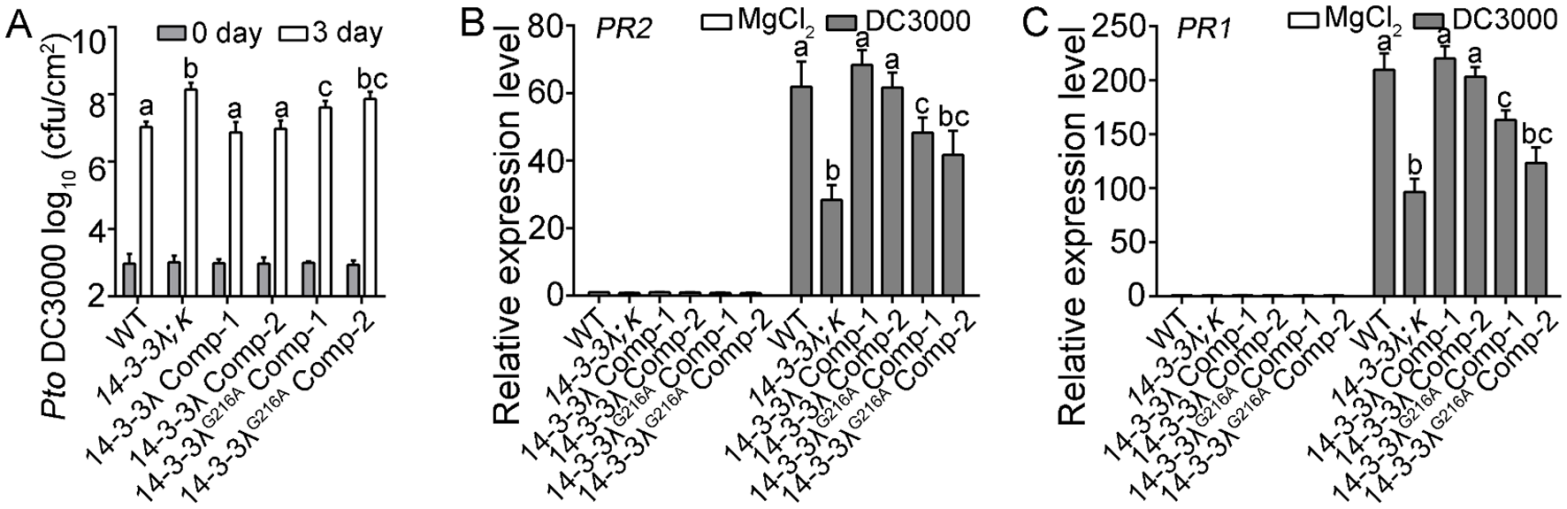
Gly216 of 14-3-3λ is required for its response to pathogen. **(A)** Bacterial growth of *Pto* DC3000 in the indicated genotypes was measured at 2 h (0 d) and 3 d post-inoculation. Values are means ± SD (n = 4). The experiment was performed three times with similar results. **(B, C)** Relative transcript levels of *PR1* **(B)** and *PR2* **(C)** in designated genotypes at 24 h after MgCl_2_ or *Pto* DC3000 treatment. Results are means ± SE (n = 3). Different letters indicate significantly different groups (1-way ANOVA and Tukey’s multiple comparison test; P < 0.05).

Since Gly216 of 14-3-3λ is a key site for its interaction with CBP60b, we investigated whether mutation at this site affects the response of 14-3-3s to pathogen. We introduced 14-3-3λ-GFP and 14-3-3λ^G216A^-GFP into the *14-3-3λ;κ* mutant and assessed their ability to complement the mutant’s response to *Pto* DC3000. Our results showed that in the 14-3-3λ-GFP transgenic lines, pathogen growth was similar to that in the wild type (**Figure 3A**), and the immune marker genes *PR1* and *PR2* were induced to levels comparable to those in the wild type (**Figures 3B, C**). In contrast, the 14-3-3λ^G216A^-GFP lines did not fully restore the mutant phenotype and instead resembled the *14-3-3λ;κ* mutant (**Figure 3**). Western blot analysis confirmed that the exogenous protein levels were relatively consistent between the *14-3-3λ-GFP* and *14-3-3λ^G216A^-GFP* transgenic lines (**Supplementary Figure 4**). These findings suggest that the response of 14-3-3s to pathogen requires Ca^2+^ binding.

### CBP60b regulates the gene expression of *14-3-3λ* and *14-3-3κ*

**Figure 4:**
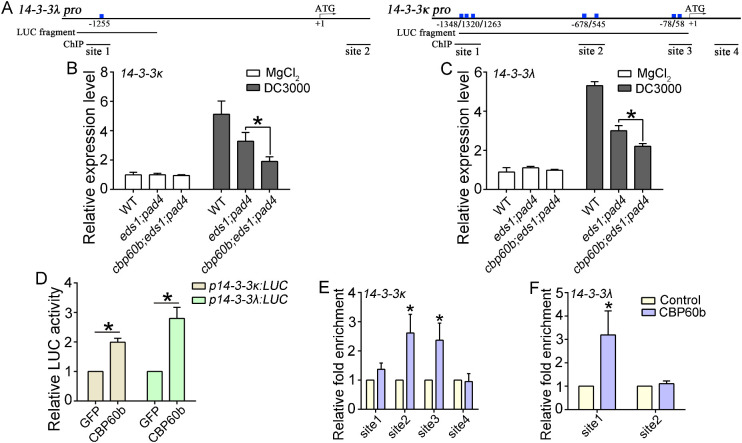
CBP60b regulates the expression of *14-3-3λ* and *14-3-3κ*. **(A)** Schematic illustration of the promoter region of *14-3-3λ* and *14-3-3κ*. +1 indicates the start of their responsive coding sequences. Blue boxes indicate the sites of G(A/T)AATT(T/G). LUC fragments and sites for ChIP PCRs are as indicated. **(B, C)** Relative transcript levels of *14-3-3κ* **(B)** and *14-3-3λ* **(C)** in designated genotypes at 24 h after MgCl_2_ or *Pto* DC3000 treatment. Results are means ± SE (n = 3). **(D)** LUC activity of *pro14-3-3κ:LUC* or *pro14-3-3λ:LUC* co-transfected with GFP or CBP60b. Values are means ± SE (n = 3). **(E, F)** ChIP-PCRs of CBP60b on *14-3-3κ* **(E)** and *14-3-3λ* **(F)**. Results are means ± SE (n = 3). Asterisks in **(B-F)** indicate significant difference (*t* test; P < 0.05).

Although 14-3-3λ and 14-3-3κ play important roles in immunity, whether their transcriptional levels are induced by pathogen remains unclear. We found that *Pto* DC3000 treatment induced the expression of *14-3-3λ* and *14-3-3κ* (**Figures 4B, C**). Promoter analysis identified putative CBP60-binding motifs in the promoter regions of *14-3-3λ* and *14-3-3κ* (**Figure 4A**). Moreover, ChIP-seq data showed that SARD1 could pull down the promoter fragment of *14-3-3λ* (Sun et al., 2015), suggesting that CBP60s may regulate *14-3-3λ* and *14-3-3κ* expression. Consistently, RT-qPCR revealed that pathogen-induced expression of *14-3-3λ* and *14-3-3κ* was lower in the *eds1;pad4;cbp60b* mutant compared to *eds1;pad4* (the *eds1;pad4* background was used to suppress the autoimmunity in *cbp60b* (Li et al., 2021)) (**Figures 4B, C**). Furthermore, LUC reporter assays using the promoter sequences of *14-3-3λ* and *14-3-3κ*, we confirmed that CBP60b induced LUC activity driven by *p14-3-3λ* and *p14-3-3κ* (**Figure 4D**). Finally, chromatin immunoprecipitation (ChIP) assays demonstrated that CBP60b directly binds to the promoter regions of *14-3-3λ* and *14-3-3κ in vivo* (**Figures 4E, F**). Taken together, these results indicate that CBP60b induces *14-3-3λ/κ* expression during immune responses.

### Loss-of-function of 14-3-3λ and 14-3-3κ partially suppress the CBP60b overexpression phenotype

**Figure 5:**
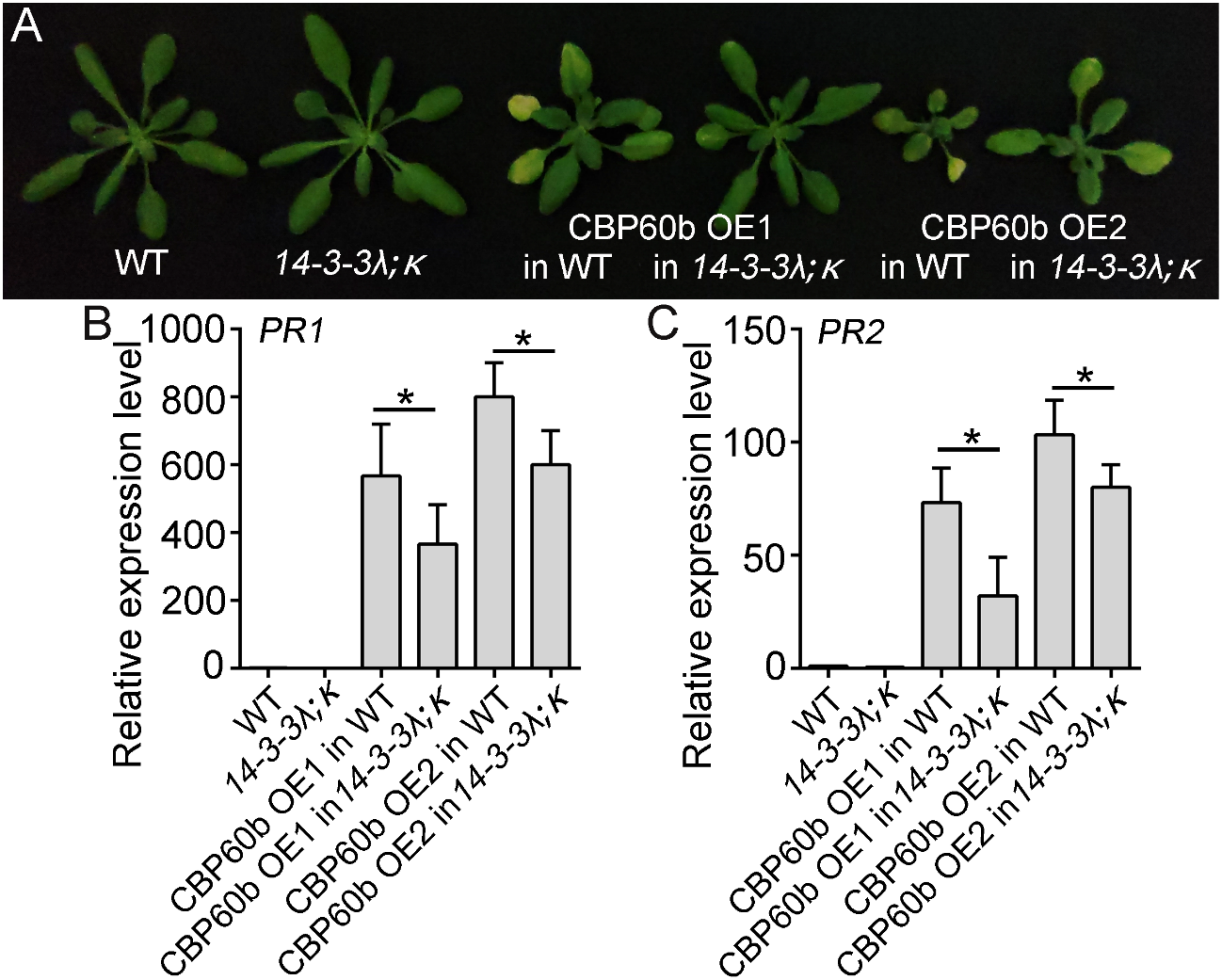
The 14-3-3λ;κ mutant partially rescues the autoimmune phenotype caused by CBP60b overexpression. **(A)** Representative growth of WT, *14-3-3λ;κ*, *CBP60b OE1* in WT or in *14-3-3λ;κ*, and *CBP60b OE2* in WT or in *14-3-3λ;κ* at 3-week-old under LD conditions. **(B, C)** Relative transcript levels of *PR1* **(B)** and *PR2* **(C)** in designated genotypes. Results are means ± SE (n = 3). Asterisks in **(B, C)** indicate significant difference (*t* test; P < 0.05).

To further investigate the regulatory relationship between CBP60b and 14-3-3s, we performed genetic experiments. We previously reported that overexpression of *CBP60b* leads to spontaneous leaf yellowing and constitutively high expression of immune genes *PR1* and *PR2* (Li et al., 2021). Through crossing, we introduced *CBP60b OE* into the *14-3-3λ;κ* mutant background. We found that in the *14-3-3λ;κ* mutant, *CBP60b OE* plants exhibited reduced leaf yellowing and decreased expression of immune genes (**Figure 5**), indicating that the autoimmune phenotype caused by *CBP60b OE* was partially suppressed. Taken together, these data suggest that the function of CBP60b in immunity depends on 14-3-3λ and 14-3-3κ.

## Discussion

**Figure 6:**
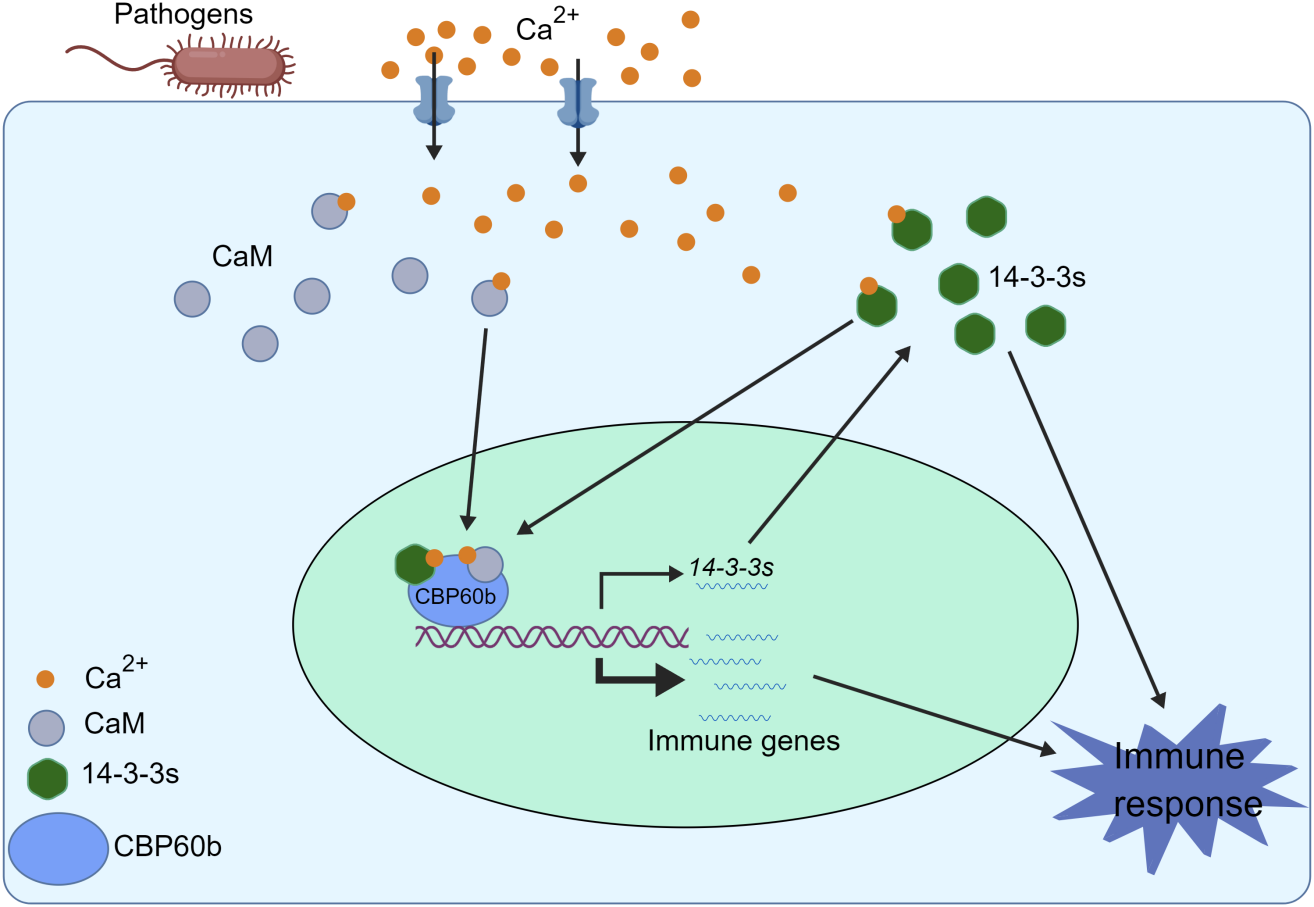
Working model: CBP60b-14-3-3λ/κ positive feedback loop integrates Ca^2+^ signaling to respond to pathogen. 14-3-3λ/κ promote the activity of CBP60b, while CBP60s mediate pathogen-induced *14-3-3λ* and *14-3-3κ* expression. CBP60b are regulated by Ca^2+^-CaM, whereas 14-3-3λ/κ require Ca^2+^ to interact with CBP60b. Thus, downstream of pathogen-induced Ca²^+^ signaling, a CBP60b-14-3-3λ/κ positive feedback loop collectively mediates pathogen resistance. The model schematic was generated using BioGDP (https://biogdp.com).

Although previous studies have well documented that CBP60b is a key transcription factor in plant immune transcriptional reprogramming, and its regulated gene subset as well as its genetic pathway have been reported (Huang et al., 2021; Li et al., 2021, Li et al., 2024), its protein interactors remain unexplored. In this study, we identified for the first time the scaffold protein 14-3-3s as interactors of CBP60b (**Figure 1**), which regulates the transcriptional activity of CBP60b (**Figure 2**). Additionally, we found that at the transcriptional level, CBP60b contributes to pathogen-induced expression of *14-3-3λ* and *14-3-3κ* (**Figure 4**). Although we did not perform an electrophoretic mobility shift assay (EMSA) to further verify the direct binding of CBP60b to the *14-3-3s* promoter *in vitro*, we are confident that the combined evidence from RT-qPCR, LUC reporter assays, and particularly the *in vivo* ChIP experiments (**Figure 4**), sufficiently demonstrates that CBP60b regulates *14-3-3s* expression. Consistently, the promoter sequence of *14-3-3λ* was also identified in the SARD1 ChIP-seq data (Sun et al., 2015), suggesting that members of the CBP60 family may be involved in regulating *14-3-3s* expression (**Figure 6**).

In this study, we demonstrate that the function of 14-3-3s in immunity requires Ca^2+^ binding. Previous research has shown that the 14-3-3λ^G216A^ mutation abolishes the Ca^2+^-binding capacity of 14-3-3λ (Yang et al., 2019). We found that 14-3-3λ^G216A^ compromised its interaction with CBP60b and the response to pathogen (**Figure 1**, **3**), suggesting that pathogen-induced elevation of Ca^2+^ levels activate the interaction between 14-3-3s and its target proteins, thereby mediating immune responses. Furthermore, the function of CBP60b requires regulation by Ca^2+^-CaM (Li et al., 2024), indicating that downstream of pathogen-induced Ca^2+^ signaling, a 14-3-3s-CBP60b positive feedback regulatory loop exists to enable a robust immune response in plant (**Figure 6**).

The roles of 14-3–3 proteins in immunity have been extensively studied, not only in Arabidopsis but also in plants such as rice, tomato, and pepper, involving processes including PTI, ETS, and ETI (Camoni et al., 2018; Sheikh et al., 2024). In this study, we identified for the first time that 14-3-3s binds to the transcription factor CBP60b in the nucleus during immune responses (**Figure 1**), thereby mediating the expression of immune-responsive genes (**Figure 2**). Members of the CBP60 family, particularly the CBP60b clade, are widely present in higher plant lineage and mediate plant immunity (Li et al., 2024). Therefore, it is plausible that the 14-3-3-CBP60b interaction module may represent a conserved mechanism mediating immunity across different plant species. This provides new insights into the study of the immune functions of 14-3–3 proteins in other plants.

While the binding motifs recognized by 14-3–3 proteins have been widely studied, it is clear that not all of their interacting partners harbor conserved motifs (Sheikh et al., 2024). In this study, we did not identify the key site in CBP60b responsible for 14-3–3 binding, despite mutating predicted sites (**Supplementary Figure 1**). However, these mutations did not affect their interaction (**Supplementary Figure 1**). Hence, further experiments—such as truncation analysis or mutational walking of CBP60b—are needed to pinpoint the critical residues mediating its interaction with 14-3-3s.

14-3–3 proteins are evolutionarily conserved molecular scaffolds in eukaryotes that mediate the localization, stability, and activity of client proteins (Sheikh et al., 2024). Arabidopsis contains 13 members of the 14-3–3 family, which exhibit high sequence similarity, suggesting extensive functional redundancy among them (van Kleeff et al., 2014; Keicher et al., 2017). Therefore, we cannot exclude the possibility—indeed, it might even be expected—that other 14-3–3 isoforms also contribute to modulating the transcriptional activity of CBP60b. This functional overlap may partly explain the relatively modest contribution of individual 14-3-3s to the activation of CBP60b downstream genes.

The precise mechanism by which 14-3–3 mediates the transcriptional activity of CBP60b in the nucleus remains to be fully elucidated. It may involve the interaction of CBP60b with subunits of the mediator complex, the recruitment of additional transcriptional regulators (An and Mou, 2013; Raya-González et al., 2025), post-translational modifications of CBP60b, or alterations in its chromatin occupancy. These represent important directions for future research.

## Data Availability

The original contributions presented in the study are included in the article/**Supplementary Material**. Further inquiries can be directed to the corresponding authors.
