# Supplementary material for: The CBP60b-14-3–3 functional module regulates immunity in arabidopsis
Source: Front Plant Sci. 2026 Aug 5;17:1916745. doi: 10.3389/fpls.2026.1916745 (PMC13486240; doi:10.3389/fpls.2026.1916745)
Supplement: Supplementary file 1 [file DataSheet1.docx]

**Supplemental Figure 1. Predicted 14-3-3 binding sites on CBP60b do not affect interaction with 14-3-3λ.**

(A) The candidate 14-3-3 binding sites on CBP60b predicted by 14-3-3-Pred (Consensus cut-off > 0.5). (B) BiFC assays showing the interaction between 14-3-3λ and CBP60b or CBP60b candidate site mutants. Bars = 50 μm.

**Supplemental Figure 2. LaCl_3_ treatment inhibits the 14-3-3-enhanced activity of CBP60b.**

(A-B) LUC activity of *proSARD1:LUC* (A) and *proEDS1:LUC* (B) co-transfected with GFP, CBP60b, or CBP60b+14-3-3λ. Add 10 μM LaCl_3_ or H2O 8 hours before harvesting protoplasts. Values are means ± SE (n = 3). Different letters indicate significantly different groups (1-way ANOVA and Tukey’s multiple comparison test; P < 0.05).

**Supplemental Figure 3. 14-3-3s do not affect the localization and protein accumulation of CBP60b**

(A-B) Representative CLSM of root meristematic zone cells from the CBP60b-GFP in WT (A) and in *14-3-3λ;κ* (B) at 5 DAG. FM4-64 staining was used to show the PM. bars = 20 μm. (C) Relative protein abundance of CBP60b-GFP in WT or in *14-3-3λ;κ* by western blot analysis. Five-week-old (5 WAG) WT and *14-3-3λ;κ* plants expressing CBP60b-GFP were infiltrated with *Pto* DC3000 (OD600 = 0.0001). Total proteins were harvested 0 or 24 h post-infiltration. Anti-Actin was used as the internal loading control in the western blot assays.

**Supplemental Figure 4. The expression level of exogenous 14-3-3λ is relatively consistent.**

Relative protein abundance of exogenous 14-3-3λ or 14-3-3λ^G216A^ in the 5 WAG indicated genotypes by western blot analysis. Anti-Actin was used as the internal loading control in the western blot assays.
